# The Time Is Now to Use Clinical Outcomes as Quality Indicators for Effective Leadership in Trauma

**DOI:** 10.5811/westjem.2016.12.33110

**Published:** 2017-02-07

**Authors:** Shahab Hajibandeh, Shahin Hajibandeh

**Affiliations:** * Queen’s Medical Centre, General Surgery Department, Nottingham, England; † Royal Blackburn Hospital, General Surgery Department, Blackburn, England

To the Editor:

We read with interest the comprehensive review by Ford et al.,^1^ which was published in August 2016 issue of the *Western Journal of Emergency Medicine*. The authors aimed to review the best available evidence regarding the effect of leadership and teamwork in trauma and resuscitation on patient care and how effective leadership can be measured.

Presence of a trauma team leader (TTL) in the trauma team is associated with positive patient outcomes in major trauma.^2^ Consistent with other authors, Ford et al.^1^ highlighted that strong leadership and teamwork can improve processes of care in trauma by improving the compliance with primary and secondary surveys. Nowadays, in major trauma centres the trauma team is lead by a designated TTL; nevertheless, what is the compliance rate with primary and secondary surveys in major trauma centres?

Compliance with the primary and secondary survey components of Advanced Trauma Life Support (ATLS) has been variable across different trauma centres. We conducted a retrospective data analysis of 93 adult trauma patients admitted to our centre, a Level I major trauma centre in England, to assess the compliance with secondary survey examinations recommended by ATLS guidelines.^3^ The compliance with secondary survey was significantly poor ranging from 1% for examination of perineum to 62% for examination of chest and limbs. In our centre the management of all trauma cases is led by designated TTLs, most of whom have instructor role in various trauma leadership training programs. So, it remains unclear why knowledge and skills developed in leadership training programs do not necessarily translate to improved clinical outcomes, such as compliance rate with trauma surveys, or missed injuries.^3^

As highlighted by Ford et al,^1^ evidence about most effective tool to measure effective leadership in trauma is lacking. The time is now to move away from non-clinical tools toward clinical outcomes to train leaders and to measure effective leadership in trauma. The current state of literature in trauma should value clinical outcomes as the most effective measures for effective leadership. Missed injuries are considered as an important issue in trauma patients and can lead to significant morbidity and even mortality; therefore, they should serve as a quality indicator in TTL performance and should remain the outcome of interest for future studies.

Who should lead the trauma team? Considering the ongoing evolution of care in trauma management and the training of nonsurgical specialties in trauma care, the composition of many trauma teams has changed. The necessity of routine surgical leadership in the resuscitative component of trauma care has been questioned by some authors due to lack of objective evidence in favour of mandatory surgical leadership of trauma teams.^4^–^6^ In view of a controversy about who should lead the trauma team, we conducted a systematic review of the literature and meta-analysis of reported outcomes associated with surgeon versus non-surgeon TTLs in management of trauma patients.^7^ Our analysis of 2,519 adult major trauma patients showed that there was no difference in survival (odds ratio [OR]: 0.82, 95% confidence interval [CI] [0.61–1.10], P=0.19) and length of stay when trauma team was led by surgeon or non-surgeon TTLs; however, fewer injuries were missed when the trauma team was led by a surgeon (OR: 0.48, 95% CI [0.25–0.92], P=0.03). However, the best available evidence was mainly from a limited number of retrospective cohort studies and high quality randomised controlled trials are required to provide more robust evidence.

In conclusion, we know from available evidence that effective leadership is associated with positive patient outcomes in major trauma; however, the current non-clinical leadership tools do not necessarily translate to improved clinical outcomes. Clinical outcomes, such as missed injuries, should be the main focus in leadership training programs, should serve as a quality indicator in TTL performance, and should remain the outcome of interest for future studies.
