# Supplementary material for: The Time Is Now to Use Clinical Outcomes as Quality Indicators for Effective Leadership in Trauma
Source: West J Emerg Med. 2017 Feb 7;18(3):331–2. doi: 10.5811/westjem.2016.12.33110 (PMC5391879; doi:10.5811/westjem.2016.12.33110)
Supplement: Related texts

# Reply: “The Time Is Now to Use Clinical Outcomes as Quality Indicators for Effective Leadership in Trauma”

**Published:** 2017-02-07

**Authors:** Elizabeth Burner, Sanjay Arora, Michael Menchine

**Affiliations:** Keck School of Medicine of the University of Southern California, Department of Emergency Medicine, Los Angeles, California

Thank you for the opportunity to respond to the letter from Drs. Shahab Hajibandeh and Shahin Hajibandeh. Their letter introduces some excellent points into this review and discussion of the role of leadership in trauma resuscitations. Identifying a leader prior to patient arrival is critical to proper team functioning and is associated with more complete primary and secondary surveys. However, non-compliance with ATLS does not necessarily equate to poor leadership skills. It is well established that some aspects of ATLS are invasive, uncomfortable and unnecessary. In fact, ATLS has been incrementally modified to reflect this reality.

The letter authors assert that clinical measures and outcomes are the future of training and studying leadership in trauma. However, while we believe that clinical outcomes are important to assess, they cannot be the only measure of leadership. We do not know if leadership behaviors have a direct impact on patient outcomes, or if they do, if other factors such as technical skill, medical knowledge, system resources or illness severity confound or mediate this proposed relationship. A physician with great leadership skills may lead the whole resuscitation in the wrong direction, to the detriment of patient care. Understanding which area of performance by a physician is flawed is important in order to correct these areas. Leadership must be directly measured in addition to clinical outcomes so that these different components can be examined individually. We identified the Leader Behavior Description Questionnaire (LBDQ) as the best validated measure of leadership based on prior literature. Furthermore, further research is needed to determine if the LBDQ or other measures of leadership correlate with improved processes of care and/or better patient outcomes.

The letter authors also bring into question who should be the identified resuscitation leader. Prior work by Leeper (2013, Journal of Trauma and Acute Care Surgery) has indicated that trauma surgeons may miss fewer minor injuries than emergency physicians, particularly in patients who have other severe injuries; however, this is a single-center study limited by a retrospective design. We believe that whether an emergency physician or a trauma surgeon is best suited to lead the initial resuscitation is not yet settled by the evidence. The ideal leader may be institution dependent and depend on the volume of significant trauma a particular center receives. In our own institution, we work collaboratively between the department of surgery and the emergency department, with the senior emergency resident serving as leader under the direct supervision of both the emergency attending and the attending trauma surgeon. We agree that regardless of who is leading the trauma resuscitation, the identified leader should have the best possible training in leadership and teamwork in order to improve processes of care. Anecdotally, there is often no formal leadership component to residency and fellowship training in the United States, and which leadership skills and behaviors are most important to teach is yet to be elucidated.

We thank the letter authors for the time they put into reading our manuscript and preparing their letter. We look forward to future studies by the Hajibandehs and other authors to clarify this exciting area.

## References

[1] Leeper WR, Leeper TJ, Vogt KN (2013). The role of trauma team leaders in missed injuries: does specialty matter?. J Trauma Acute Care Surg.
